# Successful Conservative Management of Spontaneous Greater Omental Artery Aneurysm: A Case Report

**DOI:** 10.7759/cureus.37091

**Published:** 2023-04-04

**Authors:** Mohammed Abdullah, Mustafa A Bo Khamseen, Ridha Alomran, Ali A Almohammed saleh, Mohammed A Albahrani, Mustafa A Alsaleh

**Affiliations:** 1 General Surgery, King Fahad General Hospital, Al-Ahsa, SAU; 2 General Practice, King Faisal University, Al-Ahsa, SAU; 3 College of Medicine, King Faisal University, Al-Ahsa, SAU; 4 College of Medicine, Imam Abdulrahman Bin Faisal University, Dammam, SAU; 5 Trauma, Imam Abdulrahman Bin Faisal University, Dammam, SAU

**Keywords:** interventional radiology, conservative management, computerd tomography, omental artery aneurysm, idiopathic omental bleeding

## Abstract

Omental hemorrhage is the result of a rupture of the omental vessels. Many causes have been identified to cause omental hemorrhage, which includes trauma, aneurysms, vasculitis, and neoplasms. Spontaneous omental hemorrhage is rare, and usually, patients present with a vague clinical manifestation. In this article, we present the case of a 62-year-old male patient who presented to the emergency department complaining of severe epigastric pain. He was diagnosed by enhanced computed tomography as having a great omental aneurysm and admitted to the surgical ward. The patient was treated conservatively with no apparent complications. Physicians should be made aware of the possibility of great omental bleeding even if none of the mentioned risk factors have been recognized to prevent the life-threatening complications that would follow this condition.

## Introduction

Rupture of the omental artery is considered to be a rare condition that could lead to a life-threatening hemoperitoneum [1]. There are a variety of causes that would lead to intra-abdominal bleeding, such as traumatic injury, rupture of the visceral arteries, and neoplasia, but omental pseudoaneurysms are considered to be a very rare cause [2,3]. Spontaneous rupture of omental vessels could lead to a high mortality rate if it is associated with hemoperitoneum [4]. Most of the patients that are diagnosed with spontaneous rupture of the omental artery have been treated with transcatheter arterial embolization (TAE) or surgery [5-7].

We present a case of a greater omentum artery aneurysm, which was diagnosed by abdominal contrast-enhanced computed tomography (CT) and treated successfully by conservative management only.

## Case presentation

A 62-year-old male patient with a past medical history of diabetes mellitus and hypertension presented to the emergency department (ER) complaining of severe epigastric pain for the past three days. The pain was intermittent; it started in the epigastric area and then shifted to the lower abdomen, which did not improve with analgesia and was associated with nausea, dizziness, and a presyncope attack. Negative history of abdominal trauma.

**Figure 1:**
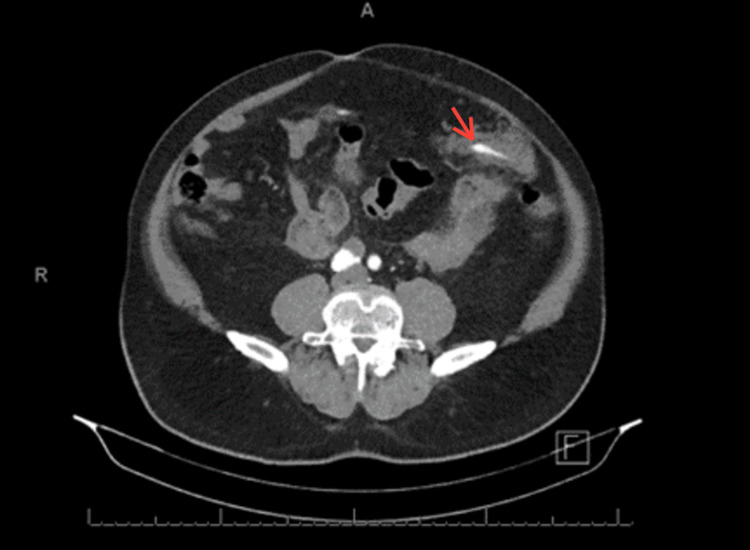
Enhanced CT scan axial view showing focal dilation of omental artery.

**Figure 2:**
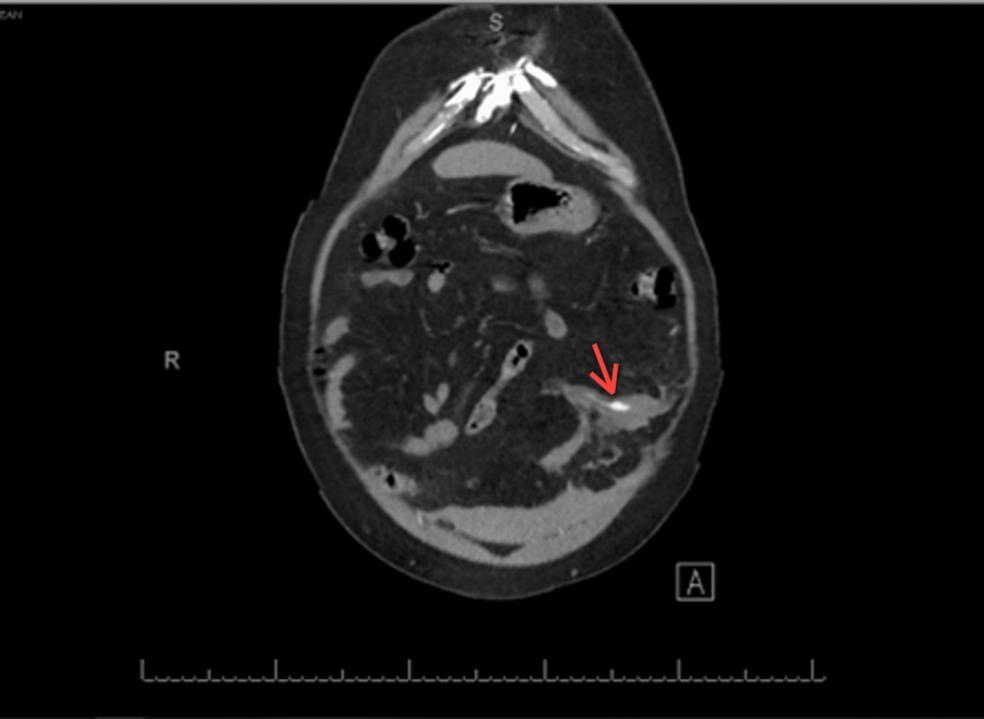
Enhanced CT scan axial view showing focal dilation of the omental artery.

On physical examination, the abdomen was soft and lax with generalized tenderness. There was no rebound tenderness or palpable mass with an audible bowel sound. Lab investigations were requested and showed that the hemoglobin level was 11.5 gm/dl, and other lab tests (LFT, RFT, and coagulation profile) were unremarkable. Abdominal enhanced computed tomography (CT) scan was requested and showed focal dilatation of the greater omentum artery surrounded by hematoma and fat stranding with no obvious active extravasation. Furthermore, it revealed the presence of moderate peri-hepatic, peri-splenic, and pelvic hemoperitoneum (Figures 1, 2).

The patient was stabilized and resuscitated in the ER with IV fluid, analgesia, and one unit of fresh frozen plasma (FFP), then admitted to the surgical ward.

During the hospital stay, the patient was kept NPO (nothing by mouth) with IV fluid, conservative management, and serial abdominal examination and investigation. Follow-up investigation showed a drop in Hb level, which reached 9.9 gm/dl and was managed by one unit of packed red blood cells and one unit of FFP. An interventional radiology opinion was requested without intervention. The patient was clinically, and laboratory improved and discharged on the fourth day of admission in good condition. In the outpatient department (OPD), follow-up was stable with no apparent complications.

## Discussion

The greater omentum gets its' blood supply from an arcade of collateral arteries branching off the left and right gastroepiploic arteries. The right gastroepiploic artery is one of two terminal branches of the gastroduodenal artery, while the left gastroepiploic artery branches off of the splenic artery [8]. Splanchnic artery aneurysms are an uncommon condition with an incidence of less than 2%. Splenic artery aneurysms are the most common, with a 60% incidence, followed by hepatic artery aneurysms at 20%, the superior mesenteric artery aneurysms at 5.5%, the celiac artery at 4%, and aneurysms of the gastric and gastro-epiploic arteries at the least frequent, at 3% [9]. Idiopathic omental bleeding is considered one of the important causes of spontaneous hemoperitoneum. It's a very serious condition with a mortality rate exceeding 30% [10]. The first known reported case of spontaneous omental bleeding occurred at Montreal General Hospital in 1918 [11]. Since then, this condition has been associated with severe trauma, malignancy, aneurysm, omental torsion, vasculitis, and a history of anticoagulant therapy or varix. Bleeding from the greater omentum without a recognized cause is referred to as idiopathic, which is a rare condition with few reported cases in the literature [2,4,8,12-14]. In our patient, there was no history of trauma, coagulopathy, or identified malignancy, and spontaneous omental bleeding was recognized by enhanced computed tomography (CT). The age of occurrence of idiopathic omental hemorrhage ranges widely from children to elderly patients, and it occurs in males more than females with a ratio of 6:1 [15].

The clinical presentation is not specific, and most of the reported cases showed that the typical signs of idiopathic omental bleeding are atypical abdominal pain, nausea and vomiting, tachycardia, and hypotensive [5,16]. Ultrasonography (US), CT scanning, and paracentesis may all be useful to establish the diagnosis. US helps facilitate hemoperitoneum detection in cases of hemodynamically unstable patients and is known to be an effective method. However, a CT scan is considered the most effective tool to diagnose idiopathic omental bleeding, which will show active arterial extravasation and hemoperitoneum. Also, it helps in the management part by localizing the bleeding site. Abdominocentesis can be another useful diagnostic tool in distinguishing the characteristics of peritoneal fluid. However, it's an invasive procedure that can lead to intestinal perforation and an abscess on the abdominal wall. So, when the patient’s condition is unstable, it may be appropriate to have a laparotomy or a laparoscopy instead [10,16]. Regardless of the underlying cause of omental hemorrhage, there are no established guidelines for the therapeutic management of omental bleeding, which is usually treated by surgical interventions, including omentectomy or ligation [4]. Furthermore, there have been multiple case reports describing transcatheter arterial embolization (TAE) as a good treatment option for omental bleeding. It's a minimally invasive procedure with a success rate reaching up to 80%. However, surgical treatment is considered the best and safest option in the presence of hemodynamic instability [16]. In the presented case, the patient was hemodynamically stable and was treated successfully with conservative management. Idiopathic omental bleeding could lead to fatal complications such as abdominal compartment syndrome, rupture, or even death [13]. Our patient is doing well in the follow-up with no apparent complications.

## Conclusions

Idiopathic omental bleeding is a rare condition with vague symptoms. Establishing the diagnosis of such a condition is extremely important avoid fatal complications. There are a variety of management options for greater omentum bleeding, and choosing between them highly depends on the patient’s condition. We described here a case of idiopathic omental bleeding that was diagnosed by enhanced CT and successfully treated conservatively with no complications.

## References

[1] Schottenfeld LE, Rubinstein H (1941). Hemorrhage and thrombosis of the omentum. Their etiology in the acute abdomen. The. American Journal of Surgery.

[2] Kimura J, Okumura K, Katagiri H, Lefor AK, Mizokami K, Kubota T (2016). Idiopathic omental hemorrhage: A case report and review of the literature. Int J Surg Case Rep.

[3] Nishiyama T, Yamada D, Oba K, Kurihara Y (2020). Left omental artery bleeding in two patients with segmental arterial mediolysis successfully isolated with coil embolization. CVIR Endovasc.

[4] Lyu YX, Cheng YX, Li T (2018). Spontaneous omental bleeding: a case report and literature review. BMC Surg.

[5] Maghrebi H, Zaiem A, Beji H (2022). Spontaneous rupture of a left omental artery aneurysm treated by transcatheter arterial embolization: A case report. Ann Med Surg (Lond).

[6] Rott G, Boecker F (2018). Segmental arterial Mediolysis of omental arteries with haemoperitoneum: case report with embolization of the left omental artery and brief review of literature. Case Rep Radiol.

[7] Stettler GR, Rauh JL, Evangelista ME, Avery MD (2022). Spontaneous rupture of omental pseudoaneurysm in a patient on systemic anticoagulation. J Surg Case Rep.

[8] Matsumoto T, Yamagami T, Morishita H (2011). Transcatheter arterial embolization for spontaneous rupture of the omental artery. Cardiovasc Intervent Radiol.

[9] Liebermann-Meffert D. (2000 The greater omentum: anatomy, embryology, and surgical applications. Surgical clinics of.

[10] Lucey BC, Varghese JC, Anderson SW, Soto JA (2007). Spontaneous hemoperitoneum: a bloody mess. Emerg Radiol.

[11] Eberts EM (1920). Case of spontaneous haemorrhage from the great omentum. Can Med Assoc J.

[12] Henry D, Satgunam S (2012). Idiopathic omental bleeding. J Surg Case Rep.

[13] Takahashi H, Adachi Y, Kasahara Y Two cases of spontaneous omental hematoma. Acta Med Kinki Univ.

[14] Ghiatas A, Fisher R CT of spontaneous haematoma of the omentum, Eur. Radiol.

[15] Takahashi M, Matsuoka Y, Yasutake T, Abe H, Sugiyama K, Oyama K (2012). Spontaneous rupture of the omental artery treated by transcatheter arterial embolization. Case Rep Radiol.

[16] Kasotakis G (2014). Spontaneous hemoperitoneum. Surg Clin North Am.

